# Validating the Nutraceutical Significance of Minor Millets by Employing Nutritional–Antinutritional Profiling

**DOI:** 10.3390/life13091918

**Published:** 2023-09-15

**Authors:** Shivani Singh Rana, Sushma Tiwari, Neha Gupta, Manoj Kumar Tripathi, Niraj Tripathi, Sangeeta Singh, Sameer S. Bhagyawant

**Affiliations:** 1 Department of Plant Molecular Biology and Biotechnology, College of Agriculture, Vijayraje Scindia Agricultural University, Gwalior 474002, Madhya Pradesh, India; shivanisinghrana885@gmail.com (S.S.R.); ng.biotech.ng@gmail.com (N.G.); 2 School of Studies in Biotechnology, Jiwaji University, Gwalior 474001, Madhya Pradesh, India; sameerbhagyawant@gmail.com; 3 Directorate of Research Services, Jawaharlal Nehru Agricultural University, Jabalpur 482004, Madhya Pradesh, India; tripathi.niraj@gmail.com; 4 National Institute of Plant Genome Research, New Delhi 110067, Delhi, India; sangeeta10mar@gmail.com

**Keywords:** minor millets, nutritional profiling, antinutritional profiling, alpha amylase and glucosidase inhibitory activities, anticancer activity

## Abstract

Millets are group of underutilized cereal crops with higher nutritional values. The present investigation used different classes of minor millets, including barnyard (sava), little (kutki), finger (ragi), kodo and foxtail millets, for evaluation of their nutritional parameters, i.e., the content of proteins, total amino acids, total sugars, insoluble fibers, soluble fibers, total dietary fibers, iron (Fe) and zinc (Zn), along with antinutritional and antioxidant parameters, viz., tannic acid, phytic acid, phenol, flavonoid, proline and 2,2-diphenyl-1-picrylhydrazyl (DPPH) radical scavenging activity. Alpha amylase and alpha glucosidase activity were also thought to elevate millets as a viable staple meal. Foxtail millet showed the maximum inhibition, with an IC_50_ value of 20.46 ± 1.80 µg mL^−1^ with respect to α-amylase. The coefficient of correlation between nutritional and antinutritional compositions showed that the starch content was significantly and positively correlated with insoluble fiber (r = 0.465) and dietary fiber (r = 0.487). Moreover, sugar was positively correlated with the phytic acid (r = 0.707), Fe and Zn (r = 0.681) contents. To determine the peptides responsible for anticancer activity, the foxtail protein was subjected to ultrafiltration; it was found that the 3 kDa fraction retained the greatest anticancer activity. Selected millet germplasm line(s) that have the best nutraceutical properties could be used in millet improvement programs.

## 1. Introduction

Millets are tiny and round-shaped grains that have extraordinary nutritional qualities with varying percentages of biomolecules such as carbohydrates, proteins, crude fiber, fat and minerals [1,2,3,4]. These underutilized crops are one of the oldest foods known to humans and were possibly the first cereal grains to be used for domestic purposes [5]. Millets belong to the Poaceae family (true grass) and are grown extensively in tropical regions. They have favorable features for surviving droughts and are highly pest resistant [6,7]. The major millets are Foxtail millet (*Setaria italica*), Finger Millet (*Eleusine oracana*) and Prosomillet or white millet (*Panicum miliaceum*), whereas the minor millets include Barnyard (*Echinochloas* pp.), Kodo (*Paspalum scrobiculatum*), Little millet (*Panicum sumatrense*), Guinea millet (*Brachiariadeflexa/Urochloa deflexa*) and Brown top millet (*Urochloa ramose/Brachiaria ramose/Panicum ramosum*) [8]. A crop such as millet that is “underutilized/neglected”, has the potential to significantly influence both production and consumption [9], as it provides a high sustainability rate and nutritional security [10]. Millets are the sixth most produced cereal grain in terms of global agricultural production and remain a staple food in many regions of world. Millets are a rich source of many vital nutrients and hence have an additional advantage for combating nutrient deficiencies in developing countries [11]. The state with the largest minor millet cultivation area is Madhya Pradesh (84,000 hectares), followed by Chhattisgarh (63,370 hectares), Uttarakhand (53,000 hectares), and others. Madhya Pradesh (84,000 hectares) has the highest production area for minor millets, followed by Chhattisgarh (63,370 hectares) and Uttarakhand (53,000 hectares). Madhya Pradesh shown the largest rise in production (74,000 tones), followed by Uttarakhand (70,970 tones) and Tamil Nadu (37,340 tons) [12].

Small millets are very important in terms of their nutritional aspects when compared with other staple crops. Minor millet species have several nutritional benefits, particularly their micronutrient and protein profiles [13]. The small millet species each represent a unique superior feature for health benefits. They are greatly preferred by diabetes and celiac disease patients because of their higher fiber content and gluten-free proteins. In the past, these millets had a huge role in helping to sustain healthy lives when there was a large amount of laborious work. With the continuous adoption of other major staple foods, the importance of millets was almost forgotten and they lost their respective value [14]. However, through festivals and traditional practices, some traditional farmers still preserve this cultural heritage, which helps us to focus on millet’s qualitative value, especially its fodder and forage value in the current era.

Owing to the great nutritional benefits and value-added products of millets, certain scientific societies have recently begun to investigate these neglected crops. Millets are being used to treat a variety of health problems, including obesity, diabetes, cardiovascular disease and cancer [15,16]. Millets have played an important role in the development of modern meals such as multigrain and gluten-free cereals. Millets are thought to play a role in lowering fat absorption and slowing sugar release (low glycemic index), owing to their high content of polyphenols and other biologically active compounds, which helps to prevent cardiovascular disease. Micronutrients, vitamins, dietary fibers, polyphenols, pigments and phytates are abundant in millets [17]. Apart from their mineral and vitamin contents, these neglected crops are comparable with other cereals such as rice and wheat in terms of protein, fat, carbohydrate and crude fiber [18]. Millets also contain phytochemicals, including phenolic acids, flavonoids and tannins, which act as natural antioxidants [11]. Millets are also renowned for containing antinutrient compounds such as alpha amylase and trypsin inhibitors, phytate and tannins. The antinutritional components in plant-based diets are being studied to learn more about their hidden health benefits. Some phenolics acids and tannins act as antioxidants and phytates protect against oxidative stress. Panwar et al. [19] evaluated five elite varieties of barnyard and finger millet growing in the northwestern Himalaya region for nutraceutical and antinutritional properties. Minor millets have been well characterized for their nutritional and nutraceutical properties; however, to date, no one has evaluated and compared five classes of minor millets including barnyard (sava), little (kutki), finger (ragi), kodo and foxtail millets for their antinutritional and nutritional compositions and their antioxidant, alpha amylase and alpha glucosidase activities. Therefore, in the current study, different classes of minor millets were investigated for their nutritional and antinutritional properties, alongside their antioxidant parameters, to categorize the millets to be used for further cultivar(s) development and as a staple food.

## 2. Materials and Method

### 2.1. Materials

The present study consisted of five classes of millet (including thirty germplasm lines), i.e., Sava millet (13), Foxtail millet (3), Ragi (2), Kodo Millet (5) and Kutki or little millet (7), that were collected from different agroclimatic regions of Madhya Pradesh, India.

### 2.2. Antinutritional Parameters

#### 2.2.1. Determination of Tannins

Dried seeds were used as a source for tannin extraction, which was performed following the method of Schandrei [20]. A total of 0.5 g of the powdered material was mixed with 75 mL of water and gently boiled for 30 min; the 100 mL volume was made by centrifuging at 2000× *g* rpm for 20 min. Then, 1 mL of the sample extract was transferred to a volumetric flask with a 100 mL capacity and 75 mL of water was added to it. A total of 10 mL of sodium carbonate solution and 5 mL of Folin–Denis reagent were diluted with 100 mL of water. After 30 min, the mixture was shaken and the absorbance was checked at 700 nm. A water blank was also prepared. If the absorbance was higher than 0.7, a proper dilution has been made. Between 0 and 100 mg of tannic acid was used to create a standard graph.

#### 2.2.2. Determination of Phytic Acid

The method suggested by Wilcox et al. [21] was employed for seed extraction with HCl (0.4 mM) for phytic acid evaluation. To obtain a clear supernatant, Chen’s reagent (3 M sulfuric acid, 2.5% ammonium molybdate, 10% ascorbic acid and deionized water in the ratio of 1:1:1:2) was utilized. The reading was recorded at a 450 nm wavelength by employing a Systronics 2203 UV-Vis spectrophotometer (Systronics, Ahmedabad, India).

#### 2.2.3. Estimation of Total Phenolic Content (TPC)

To estimate the total phenolic content, the Folin–Ciocalteu (FC) method revised by Blainski et al. [22] was employed. The reaction combination was heated at 100 °C for 1 min and stored for 2 h at room temperature. The absorbance was observed at 650 nm. The phenolic content was measured by plotting a gallic acid standard curve.

#### 2.2.4. Estimation of Total Flavonoid Content (TFC)

The total flavonoid content (TFC) was assessed by means of aluminum chloride, as described by Zou et al. [23]. The reaction was incubated for 5 min at room temperature; later, sodium hydroxide was added and the absorbance was recorded at 420 nm; quercetin was employed as the standard flavonoid.

#### 2.2.5. Determination of Proline Content

According to the procedure from Bates et al. [24], seed samples were subjected to 10 min of centrifugation at 5000× *g* rpm and aqueous sulfosalicyclic acid. Acetic acid and ninhydrin were combined in the transparent supernatant. The reaction was heated for 1 h to a temperature of 100 °C, and it was then finished by being kept in an ice bath. After mixing with toluene, it was kept in the dark for 10 min. The color intensity was measured at 520 nm and compared with standard proline.

### 2.3. Nutritional Parameters

#### 2.3.1. Extraction of Protein

Defatted seed powder (1:10) was combined with 0.5 M Tris-HCl buffer (pH 8) and 10 mM 2-mercaptoethanol as suggested by Hassan et al. [25]. The total amount protein the seeds was calculated using bovine serum albumin (BSA) as the standard (as described by Lowry et al. [26]).

#### 2.3.2. Estimation of Total Amino Acids (TAA)

Total amino acid estimation was performed by employing the ninhydrin method described by Moore and Stein [27]. Millet seed powder (100 mg) was ground in 80% ethanol and the absorbance at 570 nm was recorded.

#### 2.3.3. Estimation of Carbohydrates

The total sugar was estimated as per the method described by Dubois et al. [28]. Each seed sample (weighing about 100 mg) was mixed with 1 mL of 80% ethanol using a mortar and pestle. The solution was centrifuged at 10,000× *g* rpm for 10 min. The supernatant was then put into a tube and allowed to dry at 65 °C. The dry residue was dissolved in 1 mL of distilled water. The absorbance was recorded at a 490 nm wavelength in a spectrophotometer (Systronics, Ahmedabad, India). A standard graph of sugars was plotted for the determination of sugar content.

#### 2.3.4. Insoluble, Soluble and Total Dietary Fibers

The insoluble dietary fibers (IDFs), soluble dietary fibers (SDFs) and total dietary fibers (TDFs) were extracted by employing the method suggested by Prosky et al. [29]. Defatted seed powder was used for successive enzymatic digestion. The thermotolerant enzymes α-amylase (at 95–100 °C), protease and amyloglucosidase (at 60 °C) were used for enzymatic digestion. Precipitates were subsequently filtered, washed and dried overnight in a vacuum at a temperature of 50 °C. The insoluble and soluble dietary fibers together make up the total dietary fibers.

#### 2.3.5. Determination of Total Starch

The defatted powder was used to extract starch, adopting the method described by Annor et al. [30]. The seed powder was mixed with sodium borate buffer (12.5 mM) at pH 10, in addition to 0.5% sodium dodecyl sulfate (SDS) and Na_2_S_2_O_5_ (0.5%). The mixture was kept at room temperature for 10 min, centrifuged to separate the proteins, then washed thrice and precipitated with distilled water. The starch was suspended in water and centrifuged for 10 min. The yellow-colored particles were removed from the starch fraction.

#### 2.3.6. Atomic Absorption Spectrophotometric (AAS) Analysis

The seeds were ground to make a homogenous mixture. The mixture was treated with HNO_3_ and H_2_O_2_ (3:1) and kept on a hotplate for digestion. Whatman filter paper was used for filtration of the digested solution, and the final volume was made for metal AAS analysis by adding distilled water.

### 2.4. Antioxidant Activity

#### DPPH Radical Scavenging Activity (DPPH)

The DPPH method for scavenging activity was carried out as per the method suggested by Bondet et al. [31]. DPPH solution (0.1 mM) was added to methanolic seed solution and vigorously mixed by vortexing. The solution was stored in the dark and kept for 30 min at room temperature. The absorbance was measured at a 517 nm wavelength.

### 2.5. In Vitro Anti-Diabetes Assay

#### 2.5.1. α-Amylase Inhibition Assay

To assay α-amylase activity, the method suggested by Yu et al. [32] was employed. A 0.02 M phosphate buffer at pH 6.9 containing the α-amylase enzyme (2.5 U/mL) was pre-incubated with seeds for 10 min at 25 °C and was mixed with 1% starch in 0.02 M phosphate buffer along with 2.0 mL of 0.1 M 3, 5-dinitrosalicylic acid (DNSA). The absorbance was recorded at a 540 nm wavelength.

#### 2.5.2. α-Glucosidase Inhibition Assay

The α-glucosidase inhibitory activity was assessed using the method proposed by Yu et al. [32] with slight modifications. The 5 mM 4-nitrophenyl-β-D-glucopyranoside (pNPG) was added and kept for 10 min at 25 °C. The reaction was terminated by adding 2.0 mL of Na_2_CO_3_. The absorbance was recorded at 405 nm.

### 2.6. Characterization of Proteins

Sodium dodecyl sulfate polyacrylamide gel electrophoresis (SDS-PAGE): millet seed protein profiling was performed by using SDS-PAGE as per the method recommended by Laemmli [33]. Seed powder (25 mg) was dissolved in 1.0 mL sample buffer (0.5 M Tris-HCI pH 6.8). It was vortexed for 30 min at 5000 rpm and then centrifuged for 30 min at 5000× *g* rpm. The supernatant containing the protein was transferred to another tube. After combining 10–20 μL of the protein sample with protein dyes (SDS, glycerol, bromophenol blue and DTT), the mixture was placed in boiling water for 5–10 min. Next, the mixture was introduced onto a vertical electrophoresis apparatus at 100 V for 1.5–2 h with a 12% running gel and a 5% stacking gel. A standard protein ladder (PageRuler™ Plus Pre-stained Protein Ladder), containing 10 to 250 kDa markers, was loaded as a standard for molecular weight estimation. A staining solution, i.e., Coomassie brilliant blue R-250, was employed to visualize gels by incubating the gels in the stain for 1–2 h or overnight. The stained gels were de-stained by changing the fixing solution 1:4:5 (glacial acetic acid, distilled water, methanol) until the excess stain disappeared.

### 2.7. Purification of the Peptides from Foxtail Millet

Foxtail millet protein was purified using ultrafiltration. The foxtail millet protein was enzymatically hydrolyzed using trypsin. The foxtail millet hydrolysate was used for further analysis and fractionated via an ultrafiltration membrane system. The precise molecular mass of the purified proteins was determined using MALDI-TOF mass spectrometry. The foxtail millet hydrolysate was analyzed using an Agilent 1260 Infinity Capillary Pump coupled to a Q-TOF mass spectrometer (Agilent Technologies, Santa Clara, CA, USA). The ionization of the sample was evaluated using the relationship of mass/charge ratio (*m*/*z*), which ranges from 100 to 1100. The molecular weight of the purified peptide was determined using charged (M + H)^+1^ state analysis in the mass spectrum.

### 2.8. Sulphorhodamine B (SRB) Assay for Cytotoxicity Assessment

The cells were fixed using 10% chilled trichloroacetic acid (TCA) and kept for 1 h at 4 °C for the SRB assay following the method of Skehan et al. [34]. The SRB (0.4% *w*/*v*) was added to each well in 1% acetic acid and stored at room temperature for 30 min. The unbound SRB was detached by washing three times with 1% acetic acid. Later, 200 μL of unbuffered 10 mM tris base at pH 10.5 was used for extracting the bound stain. The absorbance was recorded at 560 nm in a microplate reader (Bio-Rad, Hercules, CA, USA).

### 2.9. Statistical Analysis

Graph pad prism (version 5) software was used for statistical analysis. The coefficients of correlation between all the nutritional and antinutritional parameters were calculated using SPSS ver.19 software. Heat map and dendrogram analyses were conducted using the “heatmap” function package in R.

## 3. Results and Discussion

Millets are high in protein, fatty acids, vitamins, minerals, dietary fiber and polyphenols, which are all vital elements [35]. The use of millets as a food supplement for increasing nutritional security and livelihoods has been documented in several studies [36,37]. Understanding the nutritional and antinutritional value of millets is critical for designing value-added goods. The current study compares the nutritional and antinutritional characteristics of thirty millet genotypes belonging of different classes that were gathered locally.

### 3.1. Total Protein and Amino Acid Content

Millets are commonly thought to be a protein source suitable for malnutrition prevention [38]. As animal protein is inadequate, costly and not sustainable, people largely depend on grain legumes for protein [39]. Thus, in the recent past, extensive studies have been conducted in order to uncover alternative protein sources. The nutritional value and quality of protein obtained from various sources are influenced by the essential amino acid composition and protein digestibility [4].

High protein and total amino acid contents accumulate in the mature seeds [40] of millets. In this context, the protein contents of the thirty millet accessions included in this investigation were assessed and found to vary and be in range of 6.1–11.4% (Table 1). The highest protein content was found in the foxtail millet genotype, whereas the lowest content was displayed by barnyard (Sava) millet. Similar to our findings, an earlier investigation on small millets conducted by Amadou et al. [35] reported a range of protein contents between 8.2 and 11.6%. However, in various other studies, finger millet was reported as a rich source of protein, with an average protein content of 10 to 11 percent [41].

Millets generally contain significant amounts of amino acids [42]. Total free amino acids were found to be in range 15.8 to 10.5 mg 100 g^−1^ in the present investigation (Table 1), with a mean value of 12.51 mg 100 g^−1^. The highest total amino acid amount was observed in little millet (Kutki-23), whereas the lowest was in barnyard millet (Sava-24).

### 3.2. Total Sugars and Starch

The major component of millets is starch, which may represent up to 70% of the total content in the seed and defines the value of millet products [35]. During the current investigation, the total carbohydrate contents varied between 71.6 and 62.4 g 100 g^−1^, with a mean amount of 65.07 g 100 g^−1^ across all the minor millet genotypes. Among all millet genotypes included in the present study, the highest values for carbohydrates were found in little millet, viz., Kutki-31, Kutki-23 and Kutki-19, kodo millet, i.e., Kodo-8 and Kodo-13 and finger millet, namely, Ragi-1 and Ragi-16. The total starch was found to be in the range 53.3 to 30.1 mg 100 g^−1^ (Table 1), with a mean value of 43.08 mg 100 g^−1^. The highest total starch was evidenced in finger millet (Ragi-1), whereas the lowest was found in the barnyard millet Sava-2. The millet genotypes with high starch contents are a good source of calories, as in the process of cooking, the starch molecules break down into a simple form of sugar [2].

### 3.3. Dietary Fibers

The average insoluble dietary fiber (IDF), soluble dietary fiber (SDF) and total dietary fiber (TDF) contents were recorded as 1.6, 14.11 and 15.71 mg 100 g^−1^, respectively, in the present investigation. The maximum TDF content was documented in the finger millet genotype Ragi-16 (21 mg 100 g^−1^); however, an average value was evidenced in the Kodo millet genotypes Kodo-8, Kodo-27, Kodo-22, Kodo-13 and Kodo-14 and the foxtail millet Fox-7 genotype. In contrast, in another report, little millet (*Panicum sumatrense* L.) and kodo millet (*Paspalum scrobiculatum*) were reported to have the highest dietary fiber contents, corresponding to 38 percent and 37 percent, respectively. In a similar study, barnyard millet was found as the richest source of crude fiber, with an average content of 12.8 g 100 g^−1^. In the past few investigations, the TDF content in finger millet was reported as being in the range 11.5 to 13.44% [43,44]. The current investigation found similar values for the TDF content in millets. Yadav et al. [45] reported 8.3% and 3.0% TDF contents in the important cereal crops wheat and rice, respectively. The TDF content values obtained during the present investigation were superior in comparison with these cereals, as the minor millet genotypes possessed higher TDF values.

### 3.4. Determination of Minerals: Fe and Zn Contents

Iron is a powerful micronutrient that is critical for human health. Both invading pathogens and mammalian cells, particularly those that are part of the immune system, require iron to maintain their function, metabolism and proliferation in infectious disorders [46]. As has recently been proven by recent studies, zinc acts as an immune booster. Zn is a micronutrient that is required for basic cell functions such as cell proliferation, differentiation and survival. Both innate and adaptive immune responses are slowed by zinc deficiency [47]. As a result of the considerable potential of these elements, researchers are focusing on and/or developing techniques to find sources that are rich in iron and zinc to boost immunity and counteract malnutrition [40]. During the present investigation, the minor millets displayed iron content in the range 20.5 (Kutki-29) to 44.6 ppm (Ragi-16). Moreover, substantial zinc contents were evidenced in five minor millet crops. The zinc content ranged between 19.2 ppm (Kutki-29) and 40.5 ppm (Fox-7 millet). The experimental values for the iron and Zn contents in millet genotypes are higher than those for several popularly consumed cereals, including wheat, rice and maize.

### 3.5. Tannic and Phytic Acid Content

Millets contain phytic acid, tannins and phenols that can contribute to antioxidant activity and are important in health, ageing and metabolic diseases [48]. In the present investigation, the tannin content was found to be in the range 3.18 mg g^−1^ to 1.85 mg g^−1^ (Table 2), with mean value of 2.44 mg g^−1^. Tannins reduce the flavor of important nutrients, mainly protein and carbohydrates, by inhibiting digestive enzymes. [49]. Tannins also have antioxidant properties that affect salivary glycoproteins [50].

The content of phytic acid in the investigated millet genotypes ranged between 6.12 and 2.11 mg g^−1^, with an average value of 3.55 mg g^−1^ (Table 2). Phosphorus is primarily stored in the form of phytic acid. By forming chelates with pro-oxidant transition metals, the phytic acid found in grains functions as an antioxidant [51]. Phytic acid has also been shown to lessen the risk of colon and breast cancers in animals, despite its reputation as an antinutrient owing to its mineral binding action [48]. Phytic acid is an essential mineral that acts as a chelator. Because monogastric animals lack the phytase enzyme in their digestive tract, absorption and bioavailability are reduced [52].

### 3.6. Total Phenolic Content and Total Flavonoid Content

Under stress conditions, plants manufacture phenolic compounds to prevent or escape from the stress conditions [53,54]. Polyphenols are the most important components in cereals in terms of antioxidant activity. During this study, soluble TPC was detected in the range 12.5 to 27.4 mg g^−1^. Verma et al. [55] reported that antioxidant ability is positively associated with the phenolic content. The role of phenolic acids has also been investigated in the treatment of diabetes. The favorable effects of phenolic acids in diabetes are attributed to the partial suppression of amylase and α-glucosidase during the enzymatic hydrolysis of complex carbohydrates, which slows glucose absorption and hence reduces postprandial blood glucose levels [56].

Flavonoids are responsible for a lower rate of chronic illnesses. Because of their free radical scavenging activities and metal ion chelating properties, they are potent antioxidants that may protect tissues from free oxygen radicals and lipid peroxidation [57]. In the present investigation, the total flavonoid content values ranged between 2.23 (foxtail millet—Fox 7) and 1.01 mg g^−1^ (finger millet—Ragi1), with a mean value of 1.45 mg g^−1^ (Table 2). Several human diseases can be controlled by eating a flavonoid-rich diet [58].

### 3.7. Proline

Proline is a stress signal that plays a vital role in plants as an antioxidant [59,60]. Proline is thought to be a plant defense mechanism that boosts reactive oxygen species (ROS) levels and balances cellular redox [61,62]. The highest average proline content across the millet genotypes was detected in Kutki-29 (0.26 mg g^−1^) and the lowest was detected in Sava-2, Sava-26, Kutki-28, Kodo-27 and Kodo-13 (0.18 mg g^−1^).

### 3.8. 2,2-Diphenyl-1-picrylhydrazyl (DPPH) Radical Scavenging Activity

Antioxidants are known to break up the free radical chain of oxidation. Antioxidants donate hydrogen atoms, resulting in a stable product [48]. The DPPH free radical scavenging activity was found to be in the range 0.62 to 0.42 mg g^−1^ (Table 2), with the highest activity found in the foxtail millet genotype Fox-7 and lowest in the little millet genotype Kutki-28; the average value across all genotypes was 0.51 mg g^−1^. DPPH is a stable free radical that reacts with antioxidants; this reaction transforms it into α, α diphenyl-β-picryl hydrazine. The antioxidant properties of cereal grains reveal disease remedial characteristics against lethal diseases such as cancer, diabetes and respiratory tract diseases [63].

### 3.9. Correlation Coefficient, Diversity Assessment and Expression Analysis among Nutritional and Antinutritional Parameters

The correlation coefficients between nutritional and antinutritional compositions signify that starch was highly significantly and positively correlated with insoluble fiber (r = 0.465) and dietary fiber (r = 0.487) at a 0.01% significance level (Table 3). Similarly, sugar was positively correlated with phytic acid (r = 0.707) and Fe with Zn (r = 0.681) at a 0.01% level of significance. Insoluble fiber was highly significantly positively correlated with dietary fiber (r = 0.991) and phytic acid (r = 0.514) at a p value of 0.01. Moreover, the zinc content was significantly correlated with tannin acid (r = 0.794) and negatively correlated with phytic acid (r = −0.506) at a 0.01 probability level (Table 3).

**Figure 1:**
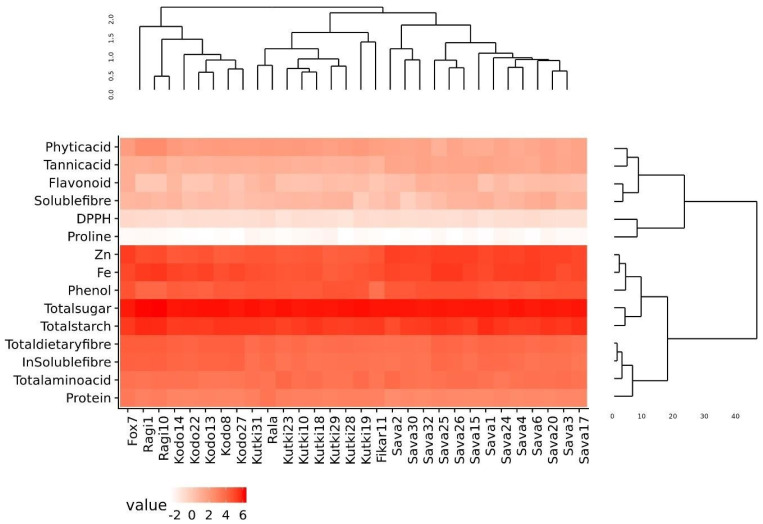
Diversity assessment and heat map of minor millet germplasm for nutritional and antinutritional parameters.

The phylogenetic analysis of the nutritional and antinutritional parameters of the minor millet germplasm lines revealed three major clusters (Figure 1). Cluster I contained the fox 7, ragi and kodo millets; cluster II contained the foxtail, kutki and fox-5 millets; and cluster III contained all the barnyard millet genotypes. The genotypes present in the same cluster exhibited similar values for nutritional and antinutritional components. The heat map shows an expression value pattern between −2 and 6, representing diverse up- and downregulated nutritional and antinutritional values in different millet classes (Figure 1).

## 4. In Vitro Anti-Diabetes Assay

### 4.1. α-Amylase and α-Glucosidase Inhibition Assay

**Figure 2:**
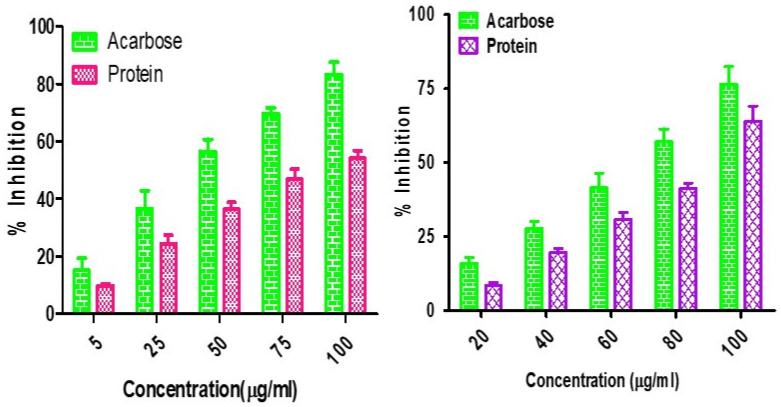
α-Amylase and α-glucosidase inhibition assay for Foxtail minor millet extracts.

α-Amylase and α-glucosidase are the enzymes responsible for preventing and causing diabetes. They are carbohydrate hydrolyzing enzymes known for their roles in dietary starch digestion and the degradation of complex oligosaccharides to monosaccharide units such as glucose during glucose level increases [64]. α-Glucosidase has a higher inhibitory activity than α-amylase in minor millets. Foxtail millet exhibited the greatest inhibition, with an IC_50_ value of 20.46 ± 1.80 µg mL^−1^ with respect to α-amylase, whereas the IC_50_ values of 21.41 ± 2.38 µg mL^−1^ for finger millet and 24.22 ± 1.68 µg mL^−1^ for little millet demonstrated almost similar results. The inhibitory activity of little millet was represented by IC_50_ values of 62.21 ± 2.80 µgmL^−1^ and 15.21 ± 4.62 µg mL^−1^ for α-amylase and α-glucosidase, respectively (Figure 2). This is in line with the report from Kwon et al. [65], who also reported that natural glucosidase inhibitors from plants had a strong inhibitory activity against glucosidase and could therefore be potentially used as an effective therapy for postprandial hyperglycemia with minimal side effects. Earlier, Italian finger millet was evaluated for the same purpose and was shown to exhibit lower IC_50_ values for the inhibition of α-glucosidase (18.07 µg mL^−1^) and α-amylase (10.56 µg mL^−1^) [66,67]. Therefore, this property could allow millets to be utilized as alternative agents with increased potency and fewer adverse effects than the existing synthetic drugs.

### 4.2. Sulforhodamine B (SRB) Assay for Cytotoxicity Assessment

**Figure 3:**
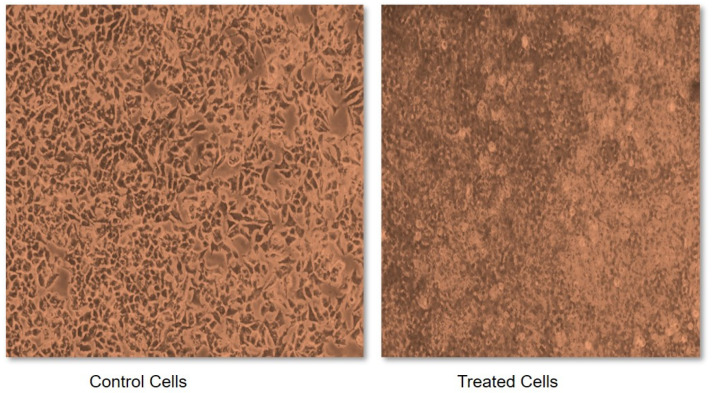
Sulforhodamine B (SRB) assay for cytotoxicity assessment of foxtail millet extracts.

Due to the characteristic inhibitory properties of α-amylase and α-glucosidase, foxtail millet was selected to test its effect on cancerous cells. This was measured in terms of an IC_50_ value, which may be defined as a quantitative measure that indicates how much of a particular inhibitory substance (for instance a drug) is needed to inhibit a given biological process or biological component by 50% in vitro. Without treatment with foxtail millet extract, the cells demonstrated abnormal structural and functional properties such as having a round shape, being shrunken, exhibiting membrane blebbing and forming apoptotic bodies (Figure 3). However, a variation in dose between 0.1 and 1.0 mg of foxtail millet extract exhibited a dose-dependent anti-proliferative activity against cancerous cells. Incubating cells with foxtail millet for a duration of 48 h gave an IC_50_ value of 0.48 mg mL^−1^ in MCF-7 cells (Figure 3). The anti-proliferative activities of foxtail millet were also observed in vitro against the human breast cancer cell line MDA and the human liver cancer cell line HepG2. Similar results were also reported for proso millet by Zhang et al. [68]. A comparable study conducted by Chandrasekara and Shahidi [69] stated that millets have the capacity to inhibit cell proliferation by 28–100% in a period of four days. Analogous results were also documented by Gupta et al. [70], who reported on the inhibitory activity of millet extract against the MCF-7 and MDA-MB-231 breast cancer cell lines. This shows the importance of foxtail millet as a potential anticancer agent. However, clinical trials to measure the safety and effectiveness of millet as a potential chemopreventive agent are needed in the future.

### 4.3. Purification of the Peptides from Foxtail Millet

**Figure 4:**
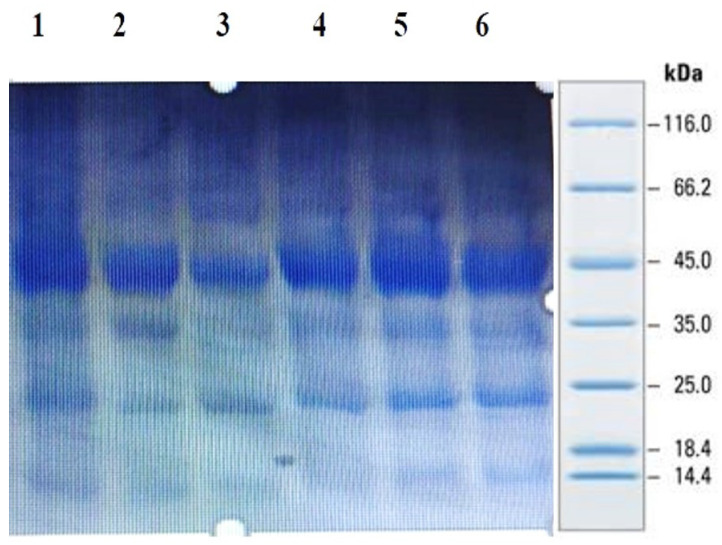
Protein profiling of minor millets in polyacrylamide gel electrophoresis (SDS PAGE).

**Figure 5:**
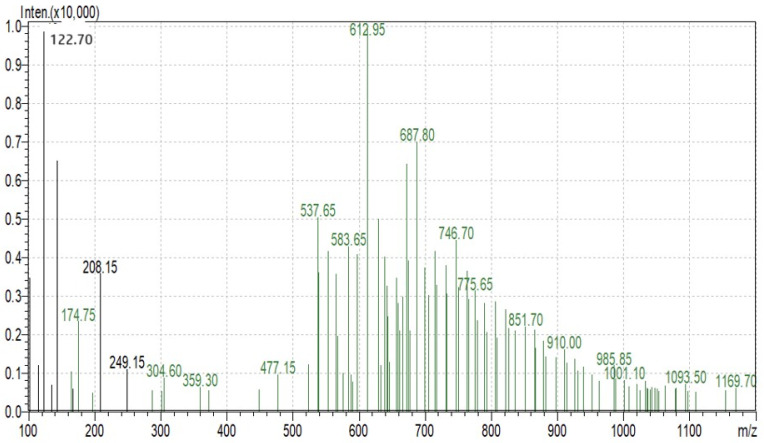
Protein fractions of Foxtail minor millet analyzed using MS.

SDS-PAGE analysis of all the millets used in the present investigation was performed to observe the proteins. Several bands were seen at 100.4, 66.2, 45.0, 35.0, 25.0, 18.4 and 14.4 kDa among all the minor millet genotypes. The minor millet lines showed vast variation in molecular weights and banding patterns for the polypeptides. Five major polypeptides, with estimated molecular weights of 66.2, 45.0, 35.0, 25.0 and 14.4 kDa, were identified (Figure 4). The molecular weight determination for the proteins was performed by employing a very sensitive technique called MALDI-TOF, which has been used in previous studies [70]. The foxtail millet protein extract was mixed with distilled water and separated using the ultrafiltration membrane system. There was a streaking in the patterns of the proteins of the fractions and the protein concentrates among the genotypes. To determine the peptides responsible for the anticancer activity, the proteins were subjected to ultrafiltration. The 3 kDa fraction was found to have the greatest anticancer activity. The molecular mass of the peptide was observed to be 612.96 Da (Figure 5). Bioactive peptides with molecular masses lower than 6000 Da possess bioactive properties [71]. The results illustrate that the lower-molecular-weight peptides facilitate the interaction between the peptide and radical species, resulting lipid inhibitory activity. This study shows that short peptide chains have the potential to cross the cellular membrane, resulting in the peptide being active in the cell.

## 5. Conclusions

Minor millets are climate-resilient crops that can fulfill the need for food and fodder and act as strong nutritional supplements. Because of this, they are considered as nutri-cereals in modern era. Peptides from millet seeds are crucial functional ingredients in the food industry. For the creation of bioactive peptides with anti-diabetes and anti-proliferative properties, foxtail millet is a crucial protein source. For their potential use and implementation in the future, investigations into the in vivo effects of millets are required. The results of the current investigation are significant for consumers, business owners and researchers.

## Figures and Tables

**Table 1:** The nutritional profiles, including protein, total amino acid, total sugar, insoluble fiber, soluble fiber, total dietary fiber, Fe and Zn, of minor millets.

S.N.	Genotypes	Protein (g100 g^−1^)	Total Amino Acid (g)	Total Starch (g 100 g^−1^)	Total Sugar (g 100 g^−1^)	Insoluble Fiber (g 100 g^−1^)	Soluble Fiber (g 100 g^−1^)	Total Dietary Fiber (g 100 g^−1^)	Fe (ppm)	Zn (ppm)
1	Sava-1	6.4 ± 0.1	12.4 ± 1.2	52.4 ± 0.9	64.2 ± 1.2	14.6 ± 2.1	2.1 ± 0.5	16.7 ± 1.4	31.2 ± 0.6	32.4 ± 0.4
2	Sava-2	6.1 ± 0.3	14.8 ± 1.3	30.1 ± 0.3	65.1 ± 1.4	11.2 ± 1.1	1.5 ± 0.5	12.7 ± 1.5	34.6 ± 0.5	38.3 ± 0.3
3	Sava-3	7.2 ± 1.1	13.2 ± 1.6	43.1 ± 0.4	62.8 ± 0.5	12.5 ± 3.1	1.7 ± 0.5	14.2 ± 1.6	28.9 ± 0.5	36.4 ± 0.3
4	Sava-4	6.4 ± 1.6	12.1 ± 0.7	39.9 ± 0.4	65.7 ± 1.4	13.6 ± 2.1	1.8 ± 0.5	15.4 ± 1.7	39.5 ± 0.7	35.2 ± 0.3
5	Sava-6	6.8 ± 0.2	12.9 ± 0.9	40.3 ± 0.5	62.4 ± 1.2	11.7 ± 1.1	2.2 ± 0.8	13.9 ± 1.8	40.8 ± 0.8	39.8 ± 0.3
6	Sava-17	7.0 ± 0.5	12.1 ± 0.4	49.5 ± 0.5	64.6 ± 1.5	10.9 ± 1.3	1.8 ± 0.9	12.7 ± 1.5	32.4 ± 0.8	33.5 ± 0.3
7	Sava-20	6.4 ± 0.8	12.7 ± 0.5	46.8 ± 0.6	64.1 ± 1.6	12.4 ± 2.3	2.5 ± 0.7	14.9 ± 0.9	36.4 ± 0.7	36.7 ± 0.3
8	Sava-24	6.9 ± 0.9	10.5 ± 0.6	44.4 ± 0.6	62.5 ± 1.4	14.5 ± 0.9	1.6 ± 0.5	16.1 ± 1.0	38.2 ± 0.6	37.2 ± 0.7
9	Sava-25	7.2 ± 1.4	12.7 ± 0.7	46.8 ± 0.8	64.8 ± 0.8	15.7 ± 2.6	1.4 ± 0.5	17.1 ± 1.6	44.2 ± 0.6	39.1 ± 0.8
10	Sava-26	6.6 ± 1.7	13.8 ± 0.8	42.8 ± 0.9	63.3 ± 1.6	14.5 ± 1.1	1.8 ± 0.7	16.3 ± 1.9	43.2 ± 0.6	38.7 ± 0.9
11	Sava-30	6.8 ± 0.2	12.5 ± 0.9	39.9 ± 0.2	64.6 ± 1.9	12.2 ± 1.1	0.8 ± 0.9	13.0 ± 1.4	32.5 ± 0.1	36.6 ± 0.4
12	Sava-32	6.3 ± 0.4	11.4 ± 0.7	41.5 ± 0.6	62.8 ± 1.7	11.6 ± 2.1	1.1 ± 0.5	12.7 ± 1.6	33.1 ± 0.4	34.8 ± 0.7
13	Sava-15	6.5 ± 0.8	13.8 ± 1.3	38.1 ± 0.7	64.0 ± 1.7	12.5 ± 2.1	1.8 ± 0.6	14.3 ± 1.7	35.6 ± 0.4	38.2 ± 0.4
14	Kutki-10	8.4 ± 0.9	12.9 ± 0.4	40.3 ± 0.3	63.9 ± 1.8	13.6 ± 1.1	1.7 ± 0.7	15.3 ± 1.8	24.5 ± 0.4	22.4 ± 0.4
15	Kutki-18	8.6 ± 0.1	13.7 ± 1.7	45.3 ± 0.5	65.3 ± 0.9	11.3 ± 2.1	1.6 ± 0.9	12.9 ± 1.1	25.7 ± 0.8	23.5 ± 0.4
16	Kutki-31	8.8 ± 0.6	12.6 ± 1.1	44.5 ± 0.6	66.7 ± 1.2	12.6 ± 1.8	1.4 ± 0.4	14.0 ± 1.4	28.2 ± 0.9	24.6 ± 0.4
17	Kutki-29	8.1 ± 1.1	11.3 ± 1.7	39.6 ± 0.7	64.1 ± 1.3	11.9 ± 0.9	1.8 ± 0.4	13.7 ± 1.6	20.5 ± 0.2	19.2 ± 0.4
18	Kutki-28	8.6 ± 1.4	11.7 ± 1.9	38.4 ± 0.8	65.8 ± 1.4	12.4 ± 2.2	1.9 ± 0.4	14.3 ± 1.7	21.6 ± 0.4	20.1 ± 0.4
19	Kutki-23	8.9 ± 1.7	15.8 ± 2.1	37.1 ± 0.2	66.7 ± 1.4	11.6 ± 2.6	1.6 ± 0.4	13.2 ± 1.7	23.4 ± 0.5	21.4 ± 0.8
20	Kutki-19	8.5 ± 2.1	14.7 ± 2.4	41.5 ± 0.3	68.2 ± 1.2	12.4 ± 1.7	0.9 ± 0.2	13.3 ± 1.7	24.8 ± 0.7	20.8 ± 0.9
21	Kodo-8	7.7 ± 2.3	10.8 ± 2.3	46.4 ± 0.5	67.2 ± 1.5	17.6 ± 1.4	1.4 ± 0.4	19.0 ± 1.8	28.4 ± 0.8	20.6 ± 0.9
22	Kodo-27	7.4 ± 1.2	11.2 ± 2.4	44.7 ± 0.7	63.4 ± 1.6	18.6 ± 1.6	1.2 ± 0.5	19.8 ± 1.3	32.4 ± 0.9	22.4 ± 0.3
23	Kodo-22	7.3 ± 1.1	12.5 ± 2.6	42.2 ± 0.6	65.6 ± 0.9	15.8 ± 1.8	1.3 ± 0.8	17.1 ± 1.3	33.4 ± 0.2	24.1 ± 0.4
24	Kodo-13	7.8 ± 1.4	10.9 ± 2.7	40.8 ± 0.8	67.1 ± 1.6	17.5 ± 1.9	1.5 ± 0.9	19.0 ± 1.3	36.7 ± 0.3	26.5 ± 0.6
25	Kodo-14	7.6 ± 1.5	12.6 ± 2.9	41.3 ± 0.9	64.2 ± 0.8	16.8 ± 1.5	1.8 ± 0.4	18.6 ± 1.4	35.6 ± 0.4	22.6 ± 0.7
26	Fox-7	10.5 ± 1.6	12.7 ± 1.1	42.8 ± 0.9	62.6 ± 1.4	19.2 ± 0.6	1.7 ± 0.3	20.9 ± 1.2	32.4 ± 0.4	40.5 ± 0.8
27	Fox-6	11.4 ± 1.7	11.6 ± 1.1	42.9 ± 0.4	63.7 ± 0.6	14.5 ± 0.7	1.5 ± 0.2	16.0 ± 1.4	26.6 ± 0.4	24.2 ± 0.9
28	Ragi-1	8.2 ± 1.8	11.8 ± 1.6	53.3 ± 0.7	70.2 ± 1.7	18.6 ± 0.8	1.8 ± 0.3	20.4 ± 1.6	41.5 ± 0.7	29.2 ± 0.2
29	Ragi-10	9.1 ± 0.9	12.6 ± 1.1	52.8 ± 0.8	71.6 ± 1.3	19.4 ± 0.9	1.6 ± 0.4	21.0 ± 1.7	44.6 ± 0.8	30.7 ± 0.4
30	Fox-5	8.2 ± 1.4	11.2 ± 1.7	42.9 ± 0.9	65.1 ± 0.4	11.8 ± 1.4	1.2 ± 0.7	13.0 ± 1.8	28.6 ± 0.9	24.8 ± 0.5

**Table 2:** Antinutritional and antioxidant profiles of the minor millets, including tannic acid, phytic acid, phenol, flavonoid, proline and DPPH.

S.N.	Genotypes	Tannic Acid (mg g^−1^)	Phytic Acid (mg g^−1^)	Phenol (mg g^−1^)	Flavonoid (mg g^−1^)	Proline (mg g^−1^)	DPPH (mg g^−1^)
1	Sava-1	3.11 ± 1.4	2.34 ± 1.7	23.6 ± 0.9	1.15 ± 2.2	0.19 ± 1.8	0.45 ± 1.6
2	Sava-2	2.93 ± 1.6	3.18 ± 0.1	22.8 ± 0.7	1.32 ± 0.4	0.18 ± 1.4	0.56 ± 2.4
3	Sava-3	2.84 ± 1.7	2.68 ± 1.1	24.8 ± 1.4	1.44 ± 0.9	0.21 ± 1.7	0.48 ± 2.7
4	Sava-4	2.64 ± 1.5	2.45 ± 1.2	23.7 ± 1.3	1.26 ± 1.5	0.22 ± 1.2	0.52 ± 2.4
5	Sava-6	2.46 ± 1.5	2.76 ± 1.3	21.4 ± 1.7	1.40 ± 1.5	0.19 ± 1.3	0.56 ± 2.7
6	Sava-17	3.07 ± 1.1	3.11 ± 0.9	24.8 ± 1.0	1.32 ± 1.7	0.21 ± 1.3	0.48 ± 2.9
7	Sava-20	3.18 ± 1.4	3.16 ± 1.4	23.4 ± 0.9	1.48 ± 1.7	0.24 ± 1.5	0.49 ± 2.7
8	Sava-24	2.84 ± 1.4	2.94 ± 1.8	21.6 ± 1.9	1.54 ± 1.8	0.25 ± 1.6	0.51 ± 2.2
9	Sava-25	2.77 ± 1.3	2.11 ± 1.9	26.9 ± 1.8	1.92 ± 1.7	0.22 ± 1.7	0.52 ± 2.1
10	Sava-26	2.91 ± 1.3	2.98 ± 1.2	27.3 ± 0.4	2.10 ± 0.4	0.18 ± 1.8	0.50 ± 1.8
11	Sava-30	2.78 ± 1.2	2.94 ± 0.4	22.7 ± 2.6	1.47 ± 0.9	0.20 ± 1.2	0.51 ± 1.9
12	Sava-32	3.12 ± 1.6	3.21 ± 0.9	26.5 ± 1.8	2.10 ± 1.9	0.19 ± 1.3	0.54 ± 2.4
13	Sava-15	2.97 ± 1.7	2.44 ± 1.5	27.4 ± 1.9	2.10 ± 1.8	0.21 ± 1.5	0.55 ± 2.1
14	Kutki-10	2.01 ± 1.8	4.12 ± 1.7	21.6 ± 1.0	1.17 ± 0.4	0.22 ± 1.6	0.51 ± 1.5
15	Kutki-18	2.11 ± 1.7	3.77 ± 1.8	22.3 ± 1.7	1.20 ± 0.1	0.23 ± 1.5	0.49 ± 1.7
16	Kutki-31	2.13 ± 1.6	3.89 ± 1.9	24.5 ± 1.9	1.56 ± 1.6	0.25 ± 1.3	0.51 ± 1.4
17	Kutki-29	1.90 ± 1.4	3.45 ± 1.3	25.6 ± 1.7	1.45 ± 1.7	0.26 ± 1.5	0.47 ± 1.2
18	Kutki-28	1.97 ± 1.2	3.89 ± 1.7	24.8 ± 1.8	1.34 ± 1.2	0.18 ± 1.3	0.42 ± 1.1
19	Kutki-23	2.16 ± 1.1	3.96 ± 1.8	22.9 ± 1.0	1.23 ± 1.2	0.20 ± 1.5	0.44 ± 1.6
20	Kutki-19	2.18 ± 1.0	4.12 ± 0.4	23.6 ± 1.3	1.44 ± 1.1	0.21 ± 1.6	0.57 ± 1.8
21	Kodo-8	2.10 ± 1.9	4.11 ± 1.0	22.7 ± 1.6	1.42 ± 0.8	0.20 ± 1.5	0.52 ± 2.2
22	Kodo-27	2.16 ± 1.1	3.87 ± 1.8	21.9 ± 1.7	1.10 ± 1.9	0.18 ± 1.3	0.49 ± 2.9
23	Kodo-22	2.11 ± 1.3	3.56 ± 1.6	19.6 ± 1.8	1.04 ± 1.6	0.19 ± 1.5	0.54 ± 2.7
24	Kodo-13	1.97 ± 1.2	3.81 ± 1.2	18.6 ± 1.2	1.06 ± 0.9	0.18 ± 1.6	0.51 ± 2.3
25	Kodo-14	1.88 ± 1.2	4.13 ± 1.3	21.4 ± 0.6	1.67 ± 1.1	0.20 ± 1.3	0.50 ± 1.9
26	Fox-7	2.24 ± 1.3	3.91 ± 1.1	26.2 ± 1.7	2.23 ± 1.9	0.21 ± 1.5	0.62 ± 1.8
27	Fox-6	2.32 ± 1.2	4.21 ± 1.6	24.1 ± 0.7	1.90 ± 1.6	0.22 ± 1.6	0.56 ± 1.9
28	Ragi-1	2.19 ± 1.4	6.12 ± 1.7	16.5 ± 1.2	1.01 ± 1.7	0.22 ± 1.3	0.57 ± 1.7
29	Ragi-10	2.47 ± 1.7	5.94 ± 1.8	15.8 ± 1.8	1.05 ± 1.8	0.21 ± 1.5	0.55 ± 1.6
30	Fox-5	1.85 ± 1.6	3.42 ± 1.4	12.5 ± 1.1	1.08 ± 1.9	0.20 ± 1.6	0.52 ± 1.2

**Table 3:** Correlation coefficients among nutritional and antinutritional parameters of the minor millet germplasm.

Correlations												
	Protein	AA	Starch	Sugar	IF	SF	DF	Fe	Zn	TA	PA	Phenol
Protein	1	−0.025	0.107	0.261	0.296	−0.148	0.275	−0.409 *	−0.473 **	−0.614 **	0.603 **	−0.153
AA		1	−0.360	0.088	−0.360	0.047	−0.353	−0.074	0.135	0.163	−0.081	0.256
Starch			1	0.342	0.465 **	0.181	0.487 **	0.320	0.019	0.125	0.326	−0.263
Sugar				1	0.339	−0.180	0.313	0.102	−0.379 *	−0.331	0.707 **	−0.518 **
IF					1	−0.041	0.991 **	0.421 *	−0.064	−0.282	0.514 **	−0.299
SF						1	0.093	0.190	0.252	0.227	−0.119	0.134
DF							1	0.445 *	−0.030	−0.250	0.496 **	−0.280
Fe								1	0.681 **	0.448 *	−0.061	−0.144
Zn									1	0.794 **	−0.506 **	0.298
TA										1	−0.550 **	0.444 *
PA											1	−0.542 **
Phenol												1

* Correlation is significant at the 0.05 level (2-tailed). ** Correlation is significant at the 0.01 level (2-tailed). AA = Amino Acid, IF = Insoluble Fiber, SF = Soluble Fiber, DF = Dietary Fiber, Fe = Iron, Zn = Zinc, TA = Tannin Acid, PA = Phytic Acid.

## Data Availability

All necessary data supporting the conclusions of this article will be available from the authors without undue reservation.
